# Separation of Isomeric Forms of Urolithin Glucuronides Using Supercritical Fluid Chromatography

**DOI:** 10.1021/acs.jafc.2c07145

**Published:** 2023-01-31

**Authors:** Ana M. Ares, Laura Toribio, Rocío García-Villalba, Jose M. Villalgordo, Yusuf Althobaiti, Francisco A. Tomás-Barberán, José Bernal

**Affiliations:** † I. U. CINQUIMA, Analytical Chemistry Group (TESEA), Faculty of Sciences, University of Valladolid, Valladolid 47011, Spain; ‡ CEBAS-CSIC, Research Group on Quality, Safety, and Bioactivity of Plant-Derived Foods, P.O. Box 164, Espinardo, Murcia 30100, Spain; § Eurofins-VillaPharma Research S.L.; Parque Tecnológico de Fuente Álamo, Fuente Álamo, Murcia E-30320, Spain; ∥ Department of Pharmacology and Toxicology, College of Pharmacy, Taif University, P.O. Box 11099, Taif 21944, Saudi Arabia

**Keywords:** chromatographic separation, supercritical fluid chromatography, glucuronides, gut microbiota metabolites, urolithins

## Abstract

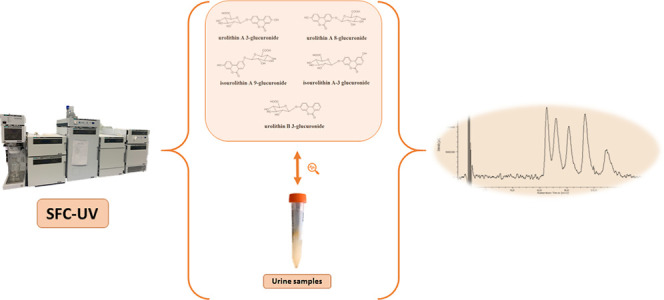

Urolithins are gut microbiota metabolites produced in humans after consuming foods containing ellagitannins and ellagic acid. Three urolithin metabotypes have been reported for different individuals depending on the final urolithins produced. After absorption, they are conjugated with glucuronic acid (phase II metabolism), and these are the main circulating metabolites in plasma and reach different tissues. Different regioisomeric isomers of urolithin glucuronides have been described. Still, their identification and quantification in humans have not been properly reported due to resolution limitations in their analysis by reversed-phase high-performance liquid chromatography. In the present study, we report a novel method for separating these isomers using supercritical fluid chromatography. With this method, urolithin A 3- and 8-glucuronide, isourolithin A 3- and 9- glucuronide, and urolithin B 3-glucuronide (8-hydroxy urolithin 3-glucuronide; 3-hydroxy urolithin 8-glucuronide; 3-hydroxyurolithin 9-glucuronide; 9-hydroxyurolithin 3-glucuronide; and urolithin 3-glucuronide) were separated in less than 15 min. The proposed method was applied to successfully analyze these metabolites in urine samples from different volunteers belonging to different metabotypes.

## Introduction

Ellagitannins and ellagic acid are polyphenols present in a wide range of plant-based foods, including berries (strawberry, raspberry, blackberry, and cranberry), grapes (muscadine), pomegranates, tropical fruits (jaboticaba and camu camu), nuts (walnuts, pecans, chestnuts, cashew, and acorns), oak-aged wines and spirits, green and black tea, herbal medicinal products, and nutraceuticals obtained from the agrifood industry (mango kernel extract and rambutan peel extract).^1^ The absorption of these polyphenols is very limited in the human body, in which they are converted in the colon by the resident microbes into urolithins.^2^ Urolithins are benzocoumarins that are much better absorbed than ellagic acid. They are conjugated by phase II metabolism to produce glucuronides and sulfates, enhancing their solubility in plasma and facilitating their excretion in urine.^3^ Urolithins have shown different biological effects, mainly demonstrated by *in vitro* and preclinical studies on different animal models.^4^ Three different urolithin metabotypes (0, A, and B), depending on the final urolithins produced by the gut microbiota (urolithin A, isourolithin A, and urolithin B), have been reported in humans after the intake of food containing ellagitannins and ellagic acid.^5^ Glucuronides of these urolithins are not commercially available, but some of the regioisomeric isomers have been recently synthesized.^7^ The analysis of the different metabolites is very relevant for describing the urolithin metabotypes and detecting differences in the phase II metabolites produced. The differences in the occurrence of these metabolites could be linked to different polymorphisms that could be related to the observed differences in the urolithinś biological effects.

In recent years, the number of research articles devoted to investigating urolithins has increased exponentially, with significant advances in the knowledge of their physiological effects,^5−7^ the metabolic pathways involved, and their presence in different biological samples (plasma, urine, feces, and tissues).^8−14^ The most widely used analytical techniques rely on high- or ultra-high-performance liquid chromatography (HPLC or UHPLC, respectively) in the reverse-phase mode employing C_18_-based columns coupled to spectrophotometric (UV–vis or DAD) and mass spectrometric (MS) detectors.^8,10,13,14^ However, the separation of isomeric forms of glucuronide urolithins using reversed-phase liquid chromatography has not been feasible as urolithin A 3- and 8-glucuronide and isourolithin A-9-glucuronide are not resolved to enable their identification and quantification eluting as a single chromatographic peak.^7,13,15^

Due to the resolution limitations of reversed-phase HPLC for analyzing urolithin glucuronides, other analytical methods should be explored to sufficiently separate these metabolites. A better peak resolution of urolithin glucuronides is essential to evaluate the differences in metabolites produced by the combined metabolism of gut microbiota, which leads to different final urolithins.^16^

One of the alternatives to HPLC could be supercritical fluid chromatography (SFC), in which the mobile phase is usually composed of a mixture of a supercritical fluid and a miscible organic solvent.^17^ The supercritical fluid, usually CO_2_, has intermediate properties between a liquid and a gas. Its density, viscosity, and diffusivity can be modified with changes in system temperature and pressure, and its polarity can be adjusted with organic modifiers. Moreover, SFC offers several advantages over HPLC, such as higher efficiencies and improved resolutions, shorter analysis times, and lower consumption of organic solvents, one of the principles of green analytical chemistry.^18^ However, it should be pointed out that, to our knowledge, no studies dealing with the SFC separation of the glucuronic derivatives of urolithin have been published, neither in biological samples nor in other matrices.

Therefore, the main goal of the present study was to investigate the potential of SFC for separating five urolithin glucuronides (urolithin A 3- and 8-glucuronide, isourolithin A 3- and 9- glucuronide, and urolithin B 3-glucuronide), paying particular attention to separate the urolithin 3-glucuronide, urolithin 8-glucuronide, and isourolithin A-9-glucuronide isomers, since it has not been ultimately achieved in any previous studies.

## Materials and Methods

### Reagents and Standards

**Figure 1:**
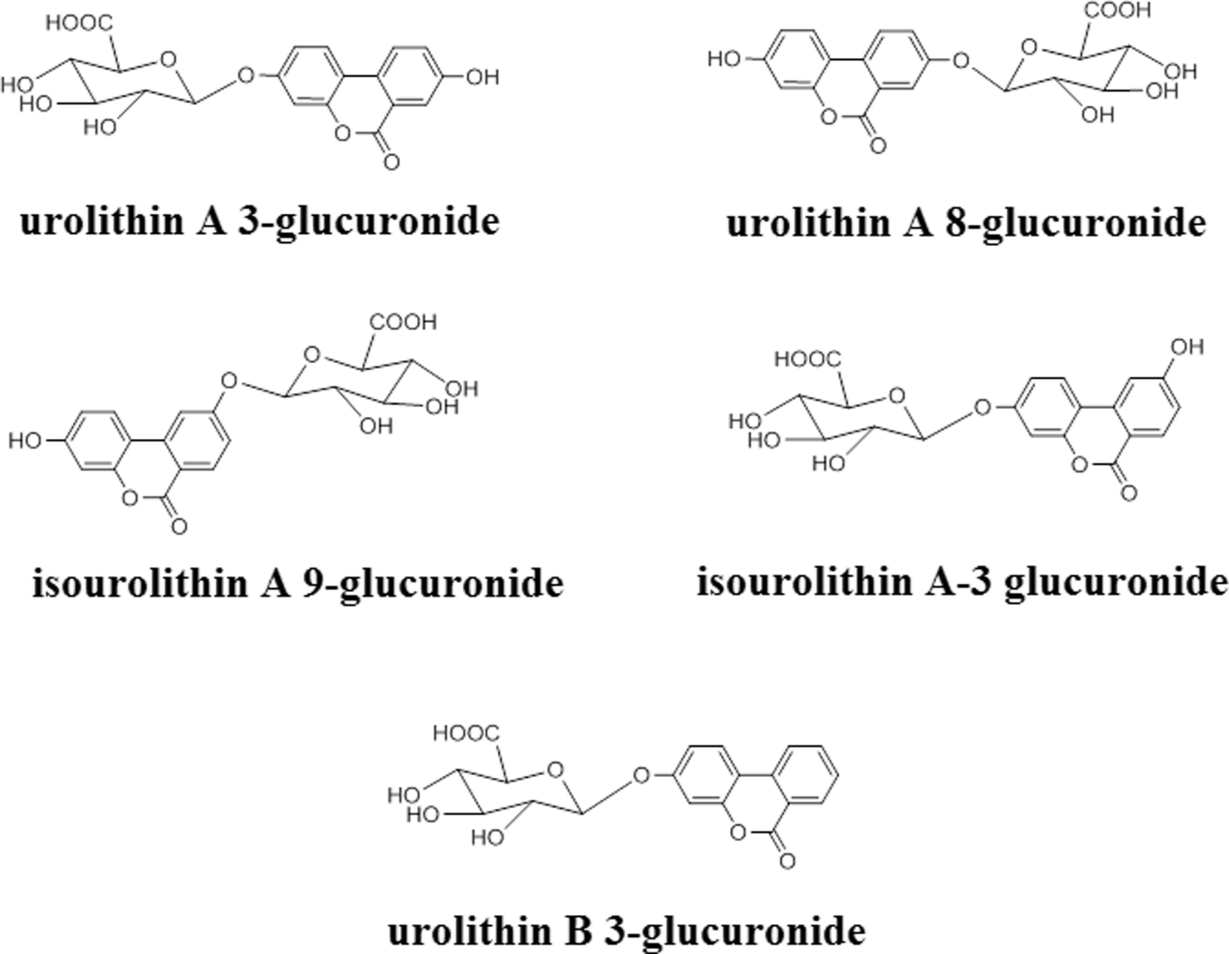
Structures of the urolithin glucuronides.

As previously reported, the different urolithin glucuronides (see structures in Figure 1) were synthesized.^6^ Standard (matrix-free) stock (≈500 mg/L) and working solutions of the studied compounds were prepared in methanol. All the organic solvents employed (methanol, acetonitrile, ethanol, isopropanol, and ethyl acetate) were of HPLC grade and obtained from LAB-SCAN (Dublin, Ireland). Trifluoroacetic acid, formic acid, acetic acid, triethylamine, and diethylamine were of analytical quality and obtained from Sigma-Aldrich (Madrid, Spain). Carbon dioxide was of SFC grade and obtained from Carburos Metálicos (Barcelona, Spain). Solid-phase extraction (SPE) cartridges Strata C_18_-E (3 mL; 500 mg; Phenomenex, Torrance, CA, USA), as well as a 10-port Visiprep vacuum manifold (Supelco, Bellefonte, PA, USA), and nylon syringe filters (17 mm, 0.45 μm; Nalgene, Rochester, NY) were employed for sample treatment.

### Sample Procurement and Treatment

Urine samples were collected from 10 healthy volunteers belonging to the three urolithin metabotypes A, B, and 0, after consuming 30 g of walnuts per day for 3 days. Urine samples were collected on the morning of the 4th day and stored refrigerated (4 °C) until analysis.^19^ Intervention was performed following the Helsinki Declaration, and informed consent was obtained from each individual. Urine samples were freeze-dried and stored at −20 °C until being analyzed. The samples were extracted and concentrated using SPE columns as previously described, with some modifications.^20^ Briefly, each urine sample (5 mL) was loaded onto a Strata C_18_-E cartridge (once conditioned with 5 mL of methanol and 5 mL of water) at about 1 mL/min employing a vacuum system. The SPE cartridge was then washed with 5 mL of water: acetic acid (99.5:0.5, v/v); the rinse was discarded, and after 5 min of drying time, the analytes were eluted with 1 mL of methanol, which was passed through a syringe filter before the SFC-UV analysis.

### SFC-UV Conditions

The SFC system was manufactured by Jasco (Tokyo, Japan). It was equipped with two pumps (4380-CO2 and 4180) for supplying the carbon dioxide and the modifier, respectively. The autosampler was a 4350 model, and the injection volume was set at 15 μL. The column was thermostated in an oven (model 4065). The pressure was controlled by a pressure regulator (model 4380), and the detector employed was a UV detector (model 4095).

An (S, S) Whelk-O 1 (150 × 4.6 mm; 3,5 μm; Regis Technologies, Morton Grove, IL, USA) column was employed for SFC analysis. The mobile phase was composed of a mixture of carbon dioxide and 0.1% (v/v) of trifluoroacetic acid in isopropanol (70:30; v/v) applied in the isocratic elution mode. The flow rate was set at 2.0 mL/min; meanwhile, injection volume, back pressure, column temperature, and detection wavelength were set at 15 μL, 130 bar, 35 °C, and 225 nm, respectively. Meanwhile, the detection wavelength was set at 225 nm.

## Results and Discussion

### Optimization of the Separation Conditions

As mentioned in the Introduction section, there are no previous methods where SFC has separated urolithin glucuronides, so it was decided to check the suitability of a series of chiral and achiral columns with different stationary phases (Table S1). Various solvents were tested as organic modifiers (methanol, ethanol, isopropanol, and acetonitrile), as well as acid (trifluoroacetic, formic, and acetic acids) and alkaline (triethylamine, diethylamine, and ethanolamine) additives or mixtures of both. Moreover, different experiments were performed for each column by varying the mobile phase’s pressure, temperature, and isocratic or gradient compositions. The initial conditions were the same for all columns. They were taken from previous experiments of the research group: mobile phase composed of CO_2_ (A) and 0.1% diethylamine in methanol (B) applied in the gradient elution mode (0–7 min, from 5% B to 60% B; 7–9 min 60% B; and 9–10, from 60% to 5% B) at a flow rate of 2.5 mL/min, 35 °C, and 100 bar. Chromatograms were registered at 225 and 305 nm and selected according to preliminary experiments (see UV–vis spectra in Supporting Information, Figure S1) and the related literature.^6^ It should also be mentioned that these experiments were performed without including urolithin B 3-glucuronide, as the main objective was to separate those compounds which usually coelute when using HPLC. The most promising results were obtained with two chiral columns, Reflect I-Cellulose C and (S, S) Whelk-O 1, both from Regis Technologies. The first column (Reflect) is based on a polysaccharide derivative (cellulose tris- 3,5-dichlorophenylcarbamate) and is suitable for many chiral compounds. At the same time, the other (Whelk-O) is a Pirkle-type column with π-electron acceptor/π-electron donor interaction abilities, which offers complementary separation capabilities to polysaccharide chiral stationary phases. More specifically, the Whelk-O column combines the π-donor (tetrahydro phenanthrene) and π-acceptor (3,5-dinitrophenyl) and hydrogen bonding sites (amide) to interact with the analytes. The preferentially bound analyte interacts *via* face-to-face π–π interactions with the dinitrophenyl moiety and hydrogen bonds with the amide function. In addition, face-to-edge π–π interactions enhance the affinity between the selector and analyte.^21^ Considering the structures of the compounds studied, they could interact through face-to-face π–π interactions between the dinitrophenyl ring of the selector and the aromatic rings of the analytes. Hydrogen-bond interactions between the NH of the chiral selector amide group and the analyte’s carbonyl group could also be possible.

The tests carried out with the other columns were not acceptable due to several reasons: (i) absence of peaks; (ii) poor peak symmetries; (iii) non-acceptable retention times; and (iv) complete coelutions (see some examples in Supporting Information, Figure S2). Thus, the optimization procedures continued with the two columns mentioned above.

### (S, S) Whelk-O 1 Column

Different solvents (methanol, acetonitrile, ethanol, and isopropanol) were tested as organic modifiers. Results showed that using methanol, the urolithin A 3- and 8-glucuronides coeluted partially, but they were baseline-separated from the isourolithin A 3- and 9- glucuronides, which were overlapped, as can be deduced from the evaluation of the chromatogram and the resolution values (Figure S3 and Table S2). Several experiments were performed using different percentages of organic modifiers, temperatures, and additives (acids: trifluoroacetic, formic, and acetic acids; bases: triethylamine, diethylamine, and ethanolamine), and the best performance was obtained when using 0.1% trifluoroacetic acid in methanol as an organic modifier (Figure S4). As can be seen, the separation between the isourolithin and urolithin isomers was better than in the first tests, especially in the case of the isourolithin isomers, whose resolution value was higher than 2 (Table S3). However, a partial coelution was also obtained between urolithin A 3-glucuronide and isourolithin A 9-glucuronide. Thus, it was decided to continue the experiments with ethanol and TFA as additives. The separation between the isourolithin and urolithin isomers was similar to that obtained using methanol. Still, the separation between urolithin A 3-glucuronide and isourolithin A 9-glucuronide improved, as can be checked by evaluating the separation parameters (Table S4).

In contrast, the resolution between the urolithin isomers was slightly worse (Figure S5 and Table S4). In the case of isopropanol, a change in selectivity for the urolithin A glucuronides was observed, but the separation was still not good enough; isopropanol does not seem to improve the results obtained with ethanol (Figure S6 and Table S5). Meanwhile, acetonitrile was discarded as most compounds were not eluted (Figure S7). After testing different mobile-phase compositions and pressure and temperature conditions with isopropanol, methanol, and ethanol, the best results were obtained with isopropanol. By reducing the composition of the organic modifier and increasing the temperature and pressure, it was possible to improve the separation of the urolithin A glucuronides. It should be specified that the retention time decreased when the pressure increased. This behavior could be explained by an increase in the density due to the higher pressure; therefore, the solvation was more significant, which increased the strength of the mobile phase.

Meanwhile, the opposite effect was observed when varying the temperature, as retention times increased. The influence of the temperature was more complex to study because it presented two opposing effects on retention. On the one hand, as the temperature increases, the density of the mobile phase decreases and, therefore, the retention increases; however, increasing the temperature favors the dissolution of the analytes in the mobile phase so that retention can de be decreased. The observed results showed that for the four different temperatures assayed (20–35 °C), the retention times of the isomer pairs increased with the temperature and the resolution (Table S6), the highest values being obtained for 35 °C. Linear van’t Hoff representations were obtained for ln*K* and the thermodynamic parameters of the separation; meanwhile, the isoelution temperatures were also calculated (see Supporting Information, Table S7). It should be noted that the values obtained are estimations from the linear correlations, and they are not exact values. In all the cases, the enthalpy (Δ*H*) and entropy (Δ*S*) variations were positive, which explained the increase in retention with temperature. Moreover, the isoelution temperatures were below the working temperature range, which means that the separation was entropically driven, and the selectivity increases with the temperature. This was also experimentally observed, and the higher resolutions were obtained at 35 °C. In order not to damage the stationary phase and considering the manufacturer’s advice, higher temperatures were not assayed. In addition, better separation between urolithin A 8-glucuronide and isourolithin A 9-glucuronide was also observed using a lower percentage of the organic modifier. It should be noted that this possibility was also evaluated with ethanol, but the separation of the urolithin A glucuronides was not improved because the effect was the opposite. Thus, as can be observed in Figure S8, the four compounds were finally separated (resolution values higher than 1 in all cases, Table S8) by using a mobile phase composed of CO_2_ and 0.1% trifluoracetic acid in isopropanol (70:30, v/v) applied in the isocratic mode at a flow rate of 2.0 mL/min, 35 °C, and 130 bar.

**Figure 2:**
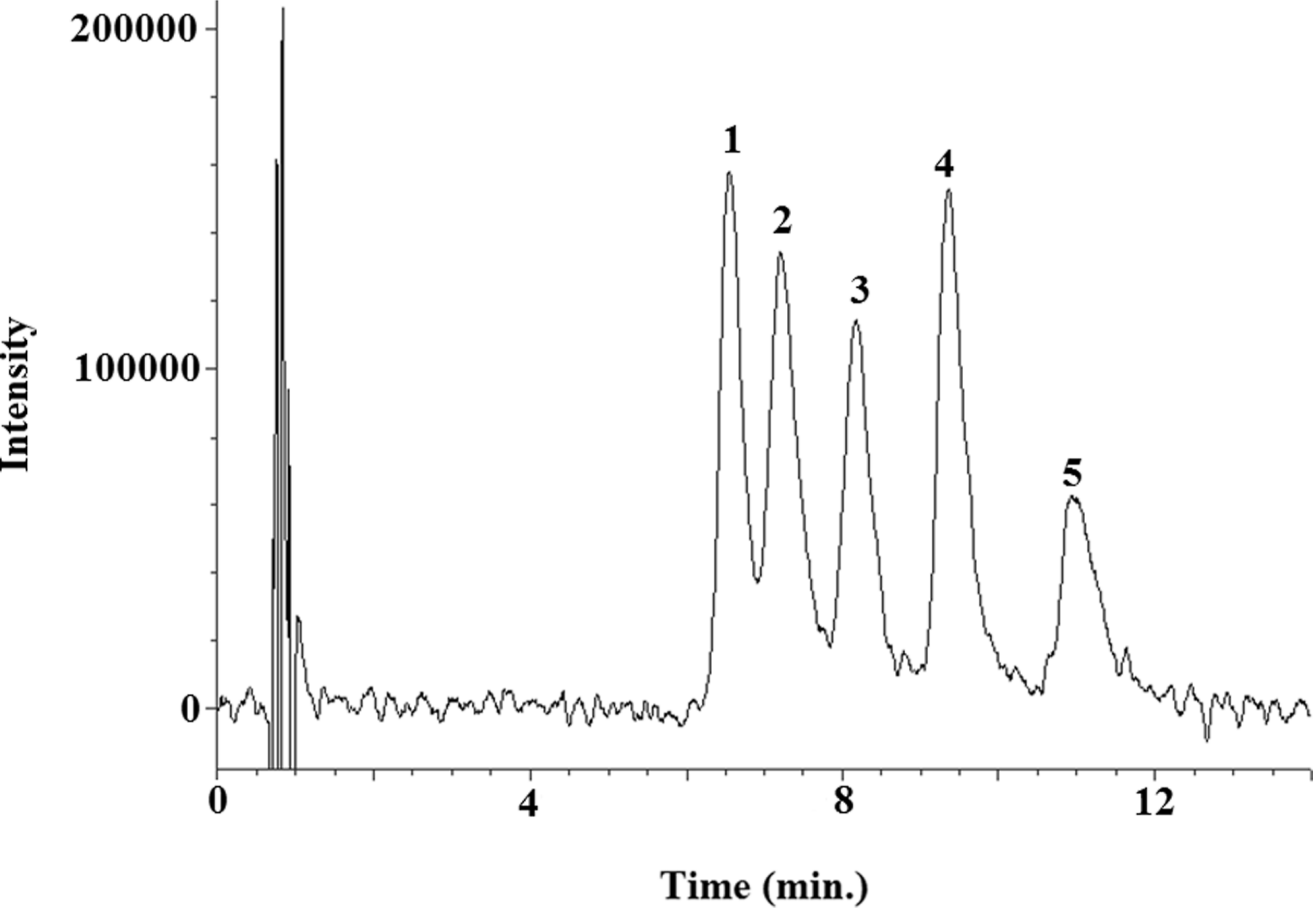
Representative SFC-UV chromatograms obtained from a mixture of urolithin glucuronide standards (100 mg/L). Chromatographic conditions are detailed in subsection **SFC-UV conditions**. 1: urolithin A 3-glucuronide; 2: urolithin A 8-glucuronide; 3: isourolithin A 9- glucuronide; 4: urolithin B 3-glucuronide; and 5: isourolithin A 3-glucuronide.

At this point, urolithin B 3-glucuronide was added to the standard mixture, as this was another relevant urolithin glucuronide in biological fluids that should also be analyzed and resolved together with the synthesized dihydroxy-urolithin glucuronides, which was injected under the selected conditions. The best possible result was obtained since this compound eluted in an empty zone of the chromatogram between two of the other compounds (see Figure 2), which was corroborated by the good resolution values (Table S9), so it was not necessary to modify any of the previously established conditions. Therefore, the five compounds were separated in less than 15 min. Finally, it should be mentioned that it was decided to register the chromatograms at 225 nm instead of the more conventional 305 nm as the peak intensities were higher.

### Reflect I-Cellulose C

Once the optimal conditions for separating the five compounds on the (S, S) Whelk-O 1 column were obtained, it was decided to test the suitability of the Reflect I-Cellulose C column. In this case, the mixture of the five compounds was used from the beginning of the optimization study. The initial conditions were optimally selected for the (S, S) Whelk-O 1 column. As can be seen in Figure S9, the results were not acceptable. The metabolites were much more retained, and the peaks were broader with poor symmetries (Table S10). Then, other mixtures of 0.1% trifluoroacetic acid with ethanol or methanol were tested. In both cases, the separation of the five compounds was not achieved due to the coelution of some of them (Figure S9). In the case of methanol, four very intense peaks with suitable shapes, symmetries, and resolutions were obtained (Table S10), although the isourolithin A glucuronides coeluted. Meanwhile, using ethanol, the separation was worse, with the central peaks corresponding to the urolithin A glucuronides being the most affected, the resolutions and shapes were worse (Table S10), and the coelution of the first two compounds was maintained. This behavior is expected as the higher the polarity of the organic modifier (methanol > ethanol > isopropanol), the greater the elution power of the mobile phase so that the analytes will be less retained. This finding is related to the organic modifier interacting with the analytes and the stationary phase, causing competition with the analyte for the active points of the stationary phase. Thus, when the adsorption phenomena of modifier molecules occur in the stationary phase, many active points of the stationary phase are blocked, and the analytes would elute before the column. Thus, methanol was selected to continue with the experiments. Afterward, the influence of the nature of the additive (basic or acidic) on the separation of the five compounds was tested. Still, in all cases, only four signals were obtained, and the separation was not as good as that obtained with trifluoracetic acid (Figure S10 and Table S11). It should be noted that when using acidic additives, shorter retention times and better peak shapes were obtained. In comparison, basic additives showed longer retention times without significant changes in peak appearance and resolution (Figure S11 and Table S12). This trend could be explained by the different ionization of the analytes and the ionizable functions of the stationary phase, which could provoke a change in the electrostatic interactions between the analyte and stationary phase. The effect of the different chromatographic parameters (temperature, pressure, and composition of the mobile phase) on the separation was also studied. The variation of the temperature and the pressure does not produce changes as significant as those obtained by modifying the mobile-phase composition. After several tests, the best separation was obtained with a mobile phase composed of CO_2_ (A) and 0.1% trifluoracetic acid in methanol (B) applied in the gradient elution mode (0–5 min, 30% B; 5–10 min, from 30% B to 40% B; and 10–11 min, from 40% B to 30% B), at a flow rate of 2.5 mL/min, 30 °C, and 120 bar. Under these conditions, the separation of the urolithin glucuronides (A-3 and A-8) and urolithin B 3-glucuronide was possible; however, isourolithin A glucuronides coeluted with each other but did not interfere with the other peaks (Figure S10 and Table S10).

In summary, the separation of the five urolithin glucuronides was achieved for the first time using SFC and the (S, S) Whelk-O 1 column, which represents a significant advance in this field of research because it would be possible to determine their occurrence and concentration individually. However, it should also be noted that the method developed with the Reflect I-Cellulose C column could be considered a valuable alternative to the selective separation of the Uro-A glucuronides and Uro-B glucuronide. In addition, the elution order of the urolithin glucuronides differed depending on the column.

### Analytical Performance of the Method

To determine the selectivity of the proposed method, a set of extracts of urine samples (*n* = 3) was injected into the chromatographic system, and the results were compared with those obtained for the individual standards of the compounds under study. It was observed that the retention times matched perfectly in all cases, with remarkable similarity in the UV spectra in standard and urine samples (data not shown). The limits of detection (LODs) and quantification (LOQs) were experimentally determined, and they were estimated to be three and ten times the signal-to-noise (S/N) ratio, respectively (Table 1). In this regard, the noise was assessed as the distribution of the response at zero analyte concentration. It should be mentioned that these values were worse than those obtained with HPLC coupled to a diode array (10–20 times higher) or MS/MS (500 times higher) detectors.^13^ The lack of sensitivity could be explained in this case by the fact that the detector available was a circular dichroism model with UV detection. Still, by incorporating both options, the detector is much less sensitive than a typical UV or a diode array detector. However, the study’s main goal was to separate the studied compounds, which was achieved, and the sensitivity could be improved by using a different detector. Calibration curves (standard in the solvent) were constructed by plotting the signal on the *y*-axis (analyte peak area) against the analyte concentration on the *x*-axis. The graphs obtained in all the calibration curves, which had a wide calibration range (LOQ-150 mg/L), were straight lines, with a coefficient of the determination values (*R*^2^) higher than 0.99 in all cases, and the residual analysis revealed a random scatter with no systematic trend (data not shown). Precision was expressed as relative standard deviation (% RSD). The experiments were performed concurrently by repeated analysis using a stock solution of the mixture of all the urolithin glucuronides (100 mg/L) and urine samples (*n* = 6; intra-day precision) or over three consecutive days (*n* = 6; inter-day precision). The obtained % RSD values for the areas and retention times were lower or equal to 15% in all cases (data not shown).

**Table 1:** Limits of Detection (LODs; mg/L), Limits of Quantification (LOQs; mg/L), and Results [Means of Triplicate Analyses; Concentration (mg/L); and % Relative Standard Deviation (% RSD) Lower Than or Equal To 15% in All Cases] of the Investigation of Human Urine Samples^a^

	urolithin A 3- glucuronide	urolithin A 8 glucuronide	isourolithin A 9- glucuronide	isourolithin A 3- glucuronide	urolithin B 3-glucuronide
LOD	6	5	7	6	7
LOQ	20	18	22	20	24
sample 1	29	32	<LOD	<LOD	<LOD
sample 2	<LOQ	<LOQ	<LOD	<LOD	<LOD
sample 3	30	<LOD	<LOD	<LOD	<LOD
sample 4	<LOQ	<LOQ	<LOD	<LOD	<LOD
sample 5	<LOQ	<LOQ	<LOD	<LOD	<LOD
sample 6	<LOQ	<LOD	36	141	<LOD
sample 7	<LOQ	<LOD	<LOQ	41	<LOQ
sample 8	21	20	<LOD	<LOD	<LOD
sample 9	23	24	<LOD	<LOD	<LOQ
sample 10	<LOD	<LOD	<LOD	<LOD	<LOD

a <LOD: under limit of detection; <LOQ: under the limit of quantification.

### Application to Human Urine Sample Analysis

**Figure 3:**
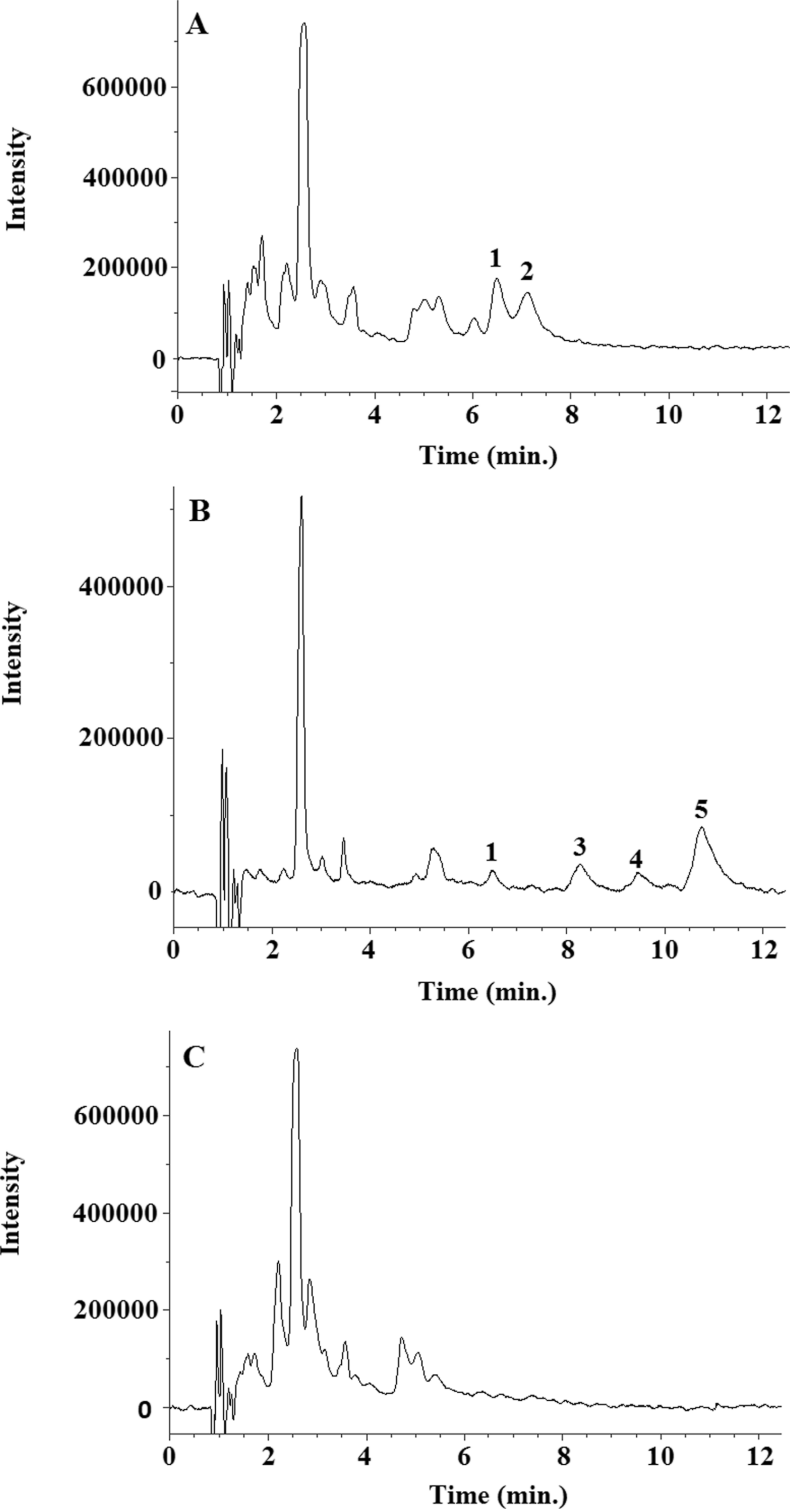
Representative SFC-UV chromatograms obtained from volunteers belonging to different urolithin metabotypes: (A) sample 8, metabotype A; (B) sample 7, metabotype B; and (C) sample 10, metabotype 0. Chromatographic conditions are detailed in subsection **SFC-UV conditions**. 1: urolithin A 3-glucuronide; 2: urolithin A 8-glucuronide; 3: isourolithin A 9- glucuronide; 4: urolithin B 3-glucuronide; and 5: isourolithin A 3-glucuronide.

The proposed SFC method was applied to analyze the five urolithin glucuronides in 10 urine samples obtained as previously described. The samples were analyzed in triplicate, and the results (mean concentration values, mg/L) are summarized in Table 1 (see chromatograms in Figure 3). Chromatographic separation of the different isomers of urolithin glucuronides was achieved in urine samples. Both isomers of urolithin A glucuronides were identified in urine samples for the first time and were present in all the volunteers with similar proportions. In metabotype B volunteers, the most common isomer was isourolithin A 3 glucuronide, although isourolithin A 9 glucuronide was also detected in some volunteers. It should be mentioned that the absence of some of the studied compounds in some of the analyzed samples could be due to the low sensitivity of the detector. Still, this issue should be solved using more sensitive detectors, especially MS/MS, in future studies.

Moreover, the chromatographic separation of the different urolithin glucuronide isomers detected in biological fluids (plasma and urine), namely, dihydroxy-urolithin (urolithin A and isourolithin A) glucuronides, is relevant as they are the primary circulating metabolites in plasma and excreted in urine in humans. Their accurate determination is important for evaluating their biological effects and the correct phenotyping of individuals into the urolithin metabotypes described above. The urolithin aglycones present in fecal samples are resolved successfully using reversed-phase HPLC analysis, which allows the adscription of individuals to the three metabotypes described.^13^ However, it is easier to obtain urine samples than fecal samples from volunteers in intervention trials, and therefore, the adscription to metabotypes analyzing urine samples is essential for most studies. There is an alternative of enzymatic hydrolysis of the glucuronide and sulfate conjugates present in plasma or urine with gluronidases and sulfatases to release the aglycones and then analyze the aglycones. This would allow the determination of the metabotypes, although relevant information regarding the conjugates present in biological fluids will be missing. The available reversed-phase HPLC method of choice for the analysis of urolithin metabolites in plasma and urine^13^ does not allow the desired resolution as three isomers (urolithin A 3-glucuronide, urolithin A-9-glucuronide, and isourolithin A 9-glucuoride)^7^ elute together in a single chromatographic peak, thus hampering the assignation of the metabotypes as metabolites characteristic of both metabotypes coelute. Regarding the interindividual variability observed in the biological effects of polyphenols,^22^ the impact of the production of specific glucuronides due to different glucuronosyl transferase genetic polymorphisms is also something that should be studied, as these metabolites can have different effects depending on the location of the glucuronide and therefore govern the occurrence of metabolites with specific free hydroxyls that can have different chemical and biochemical properties and finally potential interaction with receptors. Previous studies with human cell lines *in vitro* have shown little differences in the neuroprotective effects of glucuronide regioisomers of the same urolithin aglycone.^23^ However, differences in other biological activities cannot be discarded.

Finally, this study could represent an essential step in improving the urolithin metabotype assignment and exploring the glucuronyl transferase polymorphisms that can also affect inter-individual variations in ellagitannin metabolism and their effects on human health.

## Supporting Information Available

Stationary phases tested for the separation of the urolithin glucuronides studied; separation parameters calculated according to Purnelĺs formula; effect of temperature on the separation of the pairs of isomers with the (S, S) Whelk-O 1 column; thermodynamic parameters and isoelution temperatures (Tiso) for the pairs of isomers with the (S, S) Whelk-O 1 column; UV–vis spectra of the urolithin glucuronides; representative SFC-UV chromatograms obtained from a mixture of urolithin glucuronide standards with different columns: Lichrosphere CN, Daicel DCpak PBT, SpherexTM Diol, and Luna HILIC; representative SFC-UV chromatograms obtained from a mixture of four urolithin glucuronide standards; and representative SFC-UV chromatograms obtained from a mixture of five urolithin glucuronide standards (PDF)

## Notes

Data Availability: the data sets generated during the current study are included in this published article and the Supporting Information, or they are available from the corresponding author on reasonable request.
