# Supplementary material for: Separation of Isomeric Forms of Urolithin Glucuronides Using Supercritical Fluid Chromatography
Source: J Agric Food Chem. 2023 Jan 31;71(6):3033–9. doi: 10.1021/acs.jafc.2c07145 (PMC9936581; doi:10.1021/acs.jafc.2c07145)

SUPPORTING INFORMATION

SEPARATION OF ISOMERIC FORMS OF UROLITHIN GLUCURONIDES USING SUPERCRITICAL FLUID CHROMATOGRAPHY

Ana M. Ares,[1] Laura Toribio,[1] Rocío García-Villalba,[2] Jose M. Villalgordo,[3] Yusuf Althobaiti,[4] Francisco A. Tomás-Barberán,[2,4] José Bernal[1*]

[1] I. U. CINQUIMA, Analytical Chemistry Group (TESEA), Faculty of Sciences, University of Valladolid, 47011, Valladolid, Spain.

[2] CEBAS-CSIC, Research Group on Quality, Safety, and Bioactivity of Plant-Derived Foods. P.O. Box 164, Espinardo, Murcia 30100, Spain

[3] Eurofins-VillaPharma Research S.L.; Parque Tecnológico de Fuente Álamo. E-30320. Fuente Álamo. Murcia. Spain.

[4] Department of Pharmacology and Toxicology, College of Pharmacy, Taif University, P.O. Box 11099, Taif 21944, Saudi Arabia.

*Corresponding author: Tel# 34-983186347; e-mail: jose.bernal@uva.es

Table 1S.- Stationary phases tested for the separation of the urolithin glucuronides studied.

Column	Stationary phase	Technical specifications	Manufacturer
Achiral stationary phases			
**Silica based stationary phases**			
Luna® NH_2	Aminopropyl bonded to silica gel	150 x 4.6 mm; 5 µm	Phenomenex (Torrance, CA, USA)
Spherex™ Diol	Propanediol bonded to silica gel	250 x 4.6 mm; 5 µm	Phenomenex (Torrance, CA, USA)
Luna® Synergi Hydro	C_{18} polar endcapped bonded to silica	150 x 4.6 mm; 5 µm	Phenomenex (Torrance, CA, USA)
Luna® Omega Polar	C_{18} polar endcapped bonded to silica	100 x 2.1 mm; 3 µm	Phenomenex (Torrance, CA, USA)
Lichrosphere® CN	Cyanopropyl bonded to silica gel	150 x 4.6 mm; 5 µm	Phenomenex (Torrance, CA, USA)
Ultisil™ XB-C_{18}	C_{18}	150 x 4.6 mm; 5 µm	Welch Materials Inc. (West Haven, CT, USA)
Gemini® C_{18}	C_{18} with TMS endcapping	150 x 4.6 mm; 5 µm	Phenomenex (Torrance, CA, USA)
Kinetex® F5	Pentafluorophenyl with TMS endcapping	150 x 4.6 mm; 5 µm	Phenomenex (Torrance, CA, USA)
Daicel DCpak® PV4P	Polybutylene terephthalate coated on silica gel	250 x 4.6 mm; 5 µm	Daicel (Osaka, Japan)
Daicel DCpak® PBT	Poly(4-vinylpyridine) coated on silica gel	250 x 4.6 mm; 5 µm	Daicel (Osaka, Japan)
**Hydrophilic based stationary phases**			
Luna® HILIC	Dihydroxypropane, unbonded silica	50 x 2.0 mm; 3 µm	Phenomenex (Torrance, CA, USA)
Kinetex® HILIC	Unbonded silica	50 x 2.1 mm; 2.6 µm	Phenomenex (Torrance, CA, USA)

Table 1.- Continued.

Column	Stationary phase	Technical specifications	Manufacturer
		Chiral stationary phases	
		**Polymer based stationary phases**	
Lux® i-Amylose 3	Amylose tris(3-chloro-5-methylphenylcarbamate) bonded to silica gel	150 x 4.6 mm; 3 µm	Phenomenex (Torrance, CA, USA)
Regis Reflect® I-Cellulose C	Cellulose tris(3,5-dichlorophenylcarbamate) bonded to silica gel	150 x 4.6 mm; 3 µm	Regis Technologies (Morton Grove, IL, USA)
		**Others**	
Regis (S,S) Welk-O® 1 Kromasil	1-(3,5-Dinitrobenzamido)- 1,2,3,4,-tetrahydrophenanthrene bonded to silica gel	150 x 4.6 mm; 3.5 µm	Regis Technologies (Morton Grove, IL, USA)
Chiralpak® Zwix (+)	Quinine -derived (8S, 9R) bonded to silica gel	150 x 3.0 mm; 3 µm	Daicel (Osaka, Japan)
Astec Chirobiotic™ T2	Macrocyclic glycopeptide antibiotic teicoplanin	250 x 2.1 mm; 5 µm	Supelco (St. Louis, MO, USA)

Table 2S.- Separation parameters calculated according to Purnell´s formula referred to **Figure 3S**.

t0 = 0.76 min	Figure 3S						
**Compound**	**t_r / min**	**Rs_{1-2} (α)**	**Rs_{2-3} (α)**	**Rs_{3-4} (α)**	**Retention factor (k)**	**Symmetry Factor**	**NTP**
**1**	4.20	1.00			4.53	2.92	1672
**2**	4.68	(1.14)	0.86		5.16	2.47	1548
**3**	5.16		(1.12)	0.13	5.79	3.31	1370
**4**	5.23			(1.02)	5.88	3.23	1500

NTP: number of theoretical plates.

Table 3S.- Separation parameters calculated according to Purnell´s formula referred to **Figure 4S**.

t0 = 0.76 min	Figure 4S						
**Compound**	**t_r / min**	**Rs_{1-2} (α)**	**Rs_{2-3} (α)**	**Rs_{3-4} (α)**	**Retention factor (k)**	**Symmetry Factor**	**NTP**
**1**	3.68	0.98 (1.06)			3.84	1.13	6060
**2**	3.86		0.84 (1.05)		4.08	1.14	7124
**3**	4.02			2.53 (1.18)	4.29	1.16	7074
**4**	4.62				5.08	1.13	6060

NTP: number of theoretical plates.

Table 4S.- Separation parameters calculated according to Purnell´s formula referred to **Figure 5S**.

t0 = 0.76 min	Figure 5S						
**Compound**	**t_r / min**	**Rs_{1-2} (α)**	**Rs_{2-3} (α)**	**Rs_{3-4} (α)**	**Retention factor (k)**	**Symmetry Factor**	**NTP**
**1**	3.88	0.71			4.11	1.24	5005
**2**	4.03	(1.05)	1.60		4.30	1.31	5838
**3**	4.38		(1.11)	2.31	4.75	1.23	6416
**4**	4.98			(1.17)	5.55	1.28	5883

NTP: number of theoretical plates.

Table 5S.- Separation parameters calculated according to Purnell´s formula referred to **Figure 6S**.

**t0 = 0.76 min**	**Figure 6S**						
**Compound**	**t_r / min**	**$Rs_{1\text{-}2}$ (α)**	**$Rs_{2\text{-}3}$ (α)**	**$Rs_{3\text{-}4}$ (α)**	**Retention factor (k)**	**Symmetry Factor**	**NTP**
**1**	4.78	0.85			5.29	1.37	4413
**2**	5.07	(1.07)	0.92		5.67	1.73	3491
**3**	5.34		(1.06)	4.70	6.03	1.31	5259
**4**	7.52			(1.48)	8.89	1.79	4207

NTP: number of theoretical plates.

Table 6S.- Effect of temperature on the separation of the pairs of isomers with the (S, S) Whelk-O® 1 column. Chromatographic conditions: mobile phase composed of CO_2 and 0.1% of trifluoracetic acid in isopropanol (65:35, v/v) applied in isocratic mode at a flow rate of 2.0 mL/min and 120 bar.

Compounds	uro-A glucuronides			iso-uro-A glucuronides		
**Temperature (ºC)**	**k_1**	**k_2**	**Rs**	**k_1**	**k_2**	**Rs**
**20**	5.11	5.47	0.84	5.58	8.21	5.05
**25**	5.29	5.67	0.85	6.03	8.89	5.19
**30**	5.34	5.75	0.89	6.24	9.26	5.21
**35**	5.57	6.04	0.99	6.95	10.32	5.34

Table 7S.- Thermodynamic parameters and isoelution temperatures (T_{iso}) for the pairs of isomers with the (S, S) Whelk-O® 1 column. Chromatographic conditions: mobile phase composed of CO_2 and 0.1% of trifluoracetic acid in isopropanol (65:35, v/v) applied in isocratic mode at a flow rate of 2.0 mL/min and 120 bar.

**Compounds**	ΔH_1 (cal/mol)	ΔH_2 (cal/mol)	ΔS_1 (cal/mol K)	ΔS_2 (cal/mol K)	T_{iso} (ºC)
**uro-A glucuronides**	964.1	1106.2	6.5	7.2	-43
**iso-uro-A glucuronides**	2479.4	2597.9	11.9	13.1	-172

Table 8S.- Separation parameters calculated according to Purnell´s formula referred to **Figure 8S**.

t0 = 0.76 min	Figure 8S						
**Compound**	**t_r / min**	**Rs_{1-2} (α)**	**Rs_{2-3} (α)**	**Rs_{3-4} (α)**	**Retention factor (k)**	**Symmetry Factor**	**NTP**
**1**	6.43	1.40			7.46	1.19	2886
**2**	7.15	(1.13)	1.81		8.41	1.22	3085
**3**	8.05		(1.13)	4.62	9.54	1.35	4691
**4**	10.80			(1.38)	13.21	1.45	5134

NTP: number of theoretical plates.

Table 9S.- Separation parameters calculated according to Purnell´s formula referred to **Figure 2**.

t0 = 0.76 min	Figure 2							
**Compound**	**t_r / min**	**Rs_{1-2} (α)**	**Rs_{2-3} (α)**	**Rs_{3-4} (α)**	**Rs_{4-5} (α)**	**Retention factor (k)**	**Symmetry Factor**	**NTP**
**1**	6.30	1.20 (1.12)				7.59	1.24	2505
**2**	7.22		1.57 (1.15)			8.50	1.20	2143
**3**	8.18			2.01 (1.16)		9.76	1.30	2998
**4**	9.37				2.25 (1.18)	11.33	1.51	3966
**5**	10.93					13.38	1.40	2998

NTP: number of theoretical plates.

Table 10S.- Separation parameters calculated according to Purnell´s formula referred to **Figure 9S**.

t0 = 0.91 min	**Figure 9S-A**						
**Compound**	**t_r / min**	**$Rs_{1,2-3}$ (α)**	**Rs_{3-4} (α)**	**Rs_{4-5} (α)**	**Retention factor (k)**	**Symmetry Factor**	**NTP**
**1**	3.51	2.29			2.86	1.27	1045
**2**	4.5	(1.38)	1.43		3.95	1.35	1728
**3**	5.24		(2.64)	2.65	4.76	1.55	1639
**4**	6.66			(1.33)	6.32	1.62	2469
**t0 = 0.91 min**	**Figure 9S-B**						
**Compound**	**t_r / min**	**$Rs_{1,2-3}$ (α)**	**Rs_{3-4} (α)**	**Rs_{4-5} (α)**	**Retention factor (k)**	**Symmetry Factor**	**NTP**
**1**	4.13	2.13			3.54	1.91	1034
**2**	5.62	(1.46)	0.52		5.18	-	-
**3**	6.01		(1.08)	3.56	5.60	-	-
**4**	8.53			(1.49)	8.37	1.47	2318
**t0 = 0.91 min**	**Figure 9S-C**						
**Compound**	**t_r / min**	**Rs_{1-2} (α)**	**$Rs_{2-3,4}$ (α)**	**$Rs_{3,4-5}$ (α)**	**Retention factor (k)**	**Symmetry Factor**	**NTP**
**1**	10.58	0.92			10.63	-	-
**2**	11.73	(1.12)	0.89		11.89	-	1430
**3**	13.07		(1.12)	5.15	13.36	-	1212
**4**	19.08			(1.49)	19.97	1.33	4276

NTP: number of theoretical plates.

Table 11S.- Separation parameters calculated according to Purnell´s formula referred to **Figure 10S**.

t0 = 0.91 min	Figure 10S-A						
**Compound**	**t_r / min**	**$Rs_{1,2-3}$ (α)**	**Rs_{3-4} (α)**	**Rs_{4-5} (α)**	**Retention factor (k)**	**Symmetry Factor**	**NTP**
1	6.26	0.99			5.88	-	286
2	7.60	(1.25)	0.80		7.35	-	500
3	8.96		(1.20)	1.87	8.85	-	441
4	11.35			(1.30)	11.47	-	1250
**t0 = 0.91 min**	**Figure 10S-B**						
**Compound**	**t_r / min**	**$Rs_{1,2-3}$ (α)**	**Rs_{3-4} (α)**	**Rs_{4-5} (α)**	**Retention factor (k)**	**Symmetry Factor**	**NTP**
1	7.13	0.90			6.84	-	368
2	8.65	(1.24)	0.70		8.51	-	419
3	10.08		(1.18)	1.23	10.08	-	385
4	12.55			(1.27)	12.79	5.64	690
**t0 = 0.91 min**	**Figure 10S-C**						
**Compound**	**t_r / min**	**Rs_{1-2} (α)**	**Rs_{2-3} (α)**	**Rs_{3-4} (α)**	**Retention factor (k)**	**Symmetry Factor**	**NTP**
1	9.19	2.91			9.10	-	924
2	9.93	(1.09)	0.42		9.91	-	24335
3	10.68		(1.08)	1.14	10.74	-	575
4	12.98			(1.24;	13.26	1.82	659
5	17.89			**Rs_{4-5} (α)** 2.74 (1.41)	18.66	2.75	1597
**t0 = 0.91 min**	**Figure 10S-D**						
**Compound**	**t_r / min**	**$Rs_{1,2-3}$ (α)**	**Rs_{3-4} (α)**	**Rs_{4-5} (α)**	**Retention factor (k)**	**Symmetry Factor**	**NTP**
1	3.51	2.28			2.86	1.27	1045
2	4.50	(1.38)	1.43		3.95	1.35	1728
3	5.24		(1.21)	2.66	4.76	1.55	1639
4	6.67			(1.33)	6.33	1.62	2469

NTP: number of theoretical plates.

Table 12S.- Separation parameters calculated according to Purnell´s formula referred to **Figure 11S**.

t0 = 0.91 min	Figure 11S-A						
**Compound**	**t_r / min**	**$Rs_{1,2\text{-}3}$ (α)**	**$Rs_{3\text{-}4}$ (α)**	**$Rs_{4\text{-}5}$ (α)**	**Retention factor (k)**	**Symmetry Factor**	**NTP**
**1**	6.26	0.99			5.88	-	286
**2**	7.60	(1.25)	0.80		7.35	-	500
**3**	8.96		(1.20)	1.86	8.85	-	441
**4**	11.35			(1.30)	11.47	-	1250

t0 = 0.91 min	Figure 11S-B						
**Compound**	**t_r / min**	**$Rs_{1,2\text{-}3}$ (α)**	**$Rs_{3\text{-}4}$ (α)**	**$Rs_{4\text{-}5}$ (α)**	**Retention factor (k)**	**Symmetry Factor**	**NTP**
**1**	9.41	1.31			9.34	0.86	271
**2**	10.88	(1.17)	1.63		10.96	2.19	1492
**3**	12.87		(1.20)	3.00	13.14	1.57	1771
**4**	16.64			(1.32)	17.29	1.71	2803

t0 = 0.91 min	Figure 11S-C						
**Compound**	**t_r / min**	**$Rs_{1,2\text{-}3}$ (α)**	**$Rs_{3\text{-}4}$ (α)**	**$Rs_{4\text{-}5}$ (α)**	**Retention factor (k)**	**Symmetry Factor**	**NTP**
**1**	14.68	1.61			15.13	2.08	911
**2**	17.16	(1.18)	2.20		17.86	1.44	1989
**3**	20.25		(1.19)	3.76	21.25	1.78	3310
**4**	25.66			(1.28)	27.20	1.78	5083

t0 = 0.91 min	Figure 11S-D						
**Compound**	**t_r / min**	**$Rs_{1,2\text{-}3}$ (α)**	**$Rs_{3\text{-}4}$ (α)**	**$Rs_{4\text{-}5}$ (α)**	**Retention factor (k)**	**Symmetry Factor**	**NTP**
**1**	10.58	2.61			10.63	2.07	1020
**2**	13.96	(1.35)	1.14		14.34	-	1852
**3**	15.50		(1.12)	3.04	16.03	-	2118
**4**	19.37			(1.27)	20.29	2.15	3701

NTP: number of theoretical plates.

Figure Captions

Figure 1S.- UV-Vis spectra of the urolithin glucuronides.

Figure 2S.- Representative SFC-UV chromatograms obtained from a mixture of urolithin glucuronide standards with different columns: **A**) Lichrosphere® CN; **B**) Daicel DCpak® PBT; **C**) Spherex™ Diol; **D**) Luna® HILIC.

Figure 3S.- Representative SFC-UV chromatograms obtained from a mixture of four urolithin glucuronide standards (100 mg/L; 1.- urolithin A 8-glucuronide; 2.- urolithin A 3-glucuronide 3.- isourolithin A 9- glucuronide; 4.- isourolithin A 3-glucuronide) using the Regis (S,S) Welk-O® 1 Kromasil column and the initial conditions (see ***Optimization of the separation conditions*** subsection).

Figure 4S.- Representative SFC-UV chromatograms obtained from a mixture of four urolithin glucuronide standards (100 mg/L; 1.- urolithin A 8-glucuronide; 2.- urolithin A 3-glucuronide 3.- isourolithin A 9- glucuronide; 4.- isourolithin A 3-glucuronide) using the Regis (S,S) Welk-O® 1 Kromasil column and 0.1% (v/v) trifluoroacetic acid in methanol as organic modifier (see ***Optimization of the separation conditions*** subsection).

Figure 5S.- Representative SFC-UV chromatograms obtained from a mixture of four urolithin glucuronide standards (100 mg/L; 1.- urolithin A 8-glucuronide; 2.- urolithin A 3-glucuronide 3.- isourolithin A 9- glucuronide; 4.- isourolithin A 3-glucuronide) using the Regis (S,S) Welk-O® 1 Kromasil column and 0.1% (v/v) trifluoroacetic acid in ethanol as organic modifier (see ***Optimization of the separation conditions*** subsection).

Figure 6S.- Representative SFC-UV chromatograms obtained from a mixture of four urolithin glucuronide standards (100 mg/L; 1.- urolithin A 3-glucuronide; 2.- urolithin A 8-glucuronide 3.- isourolithin A 9- glucuronide; 4.- isourolithin A 3-glucuronide) using the Regis (S,S) Welk-O® 1 Kromasil column and 0.1% (v/v) trifluoroacetic acid in isopropanol as organic modifier (see ***Optimization of the separation conditions*** subsection).

Figure 7S.- Representative SFC-UV chromatograms obtained from a mixture of four urolithin glucuronide standards (100 mg/L; isourolithin A 9- glucuronide) using the Regis (S,S) Welk-O® 1 Kromasil column and 0.1% (v/v) trifluoroacetic acid in acetonitrile as organic modifier (see ***Optimization of the separation conditions*** subsection).

Figure 8S.- Representative SFC-UV chromatograms obtained from a mixture of four urolithin glucuronide standards (100 mg/L; 1.- urolithin A 3-glucuronide; 2.- urolithin A 8-glucuronide 3.- isourolithin A 9- glucuronide; 4.- isourolithin A 3-glucuronide) using the Regis (S,S) Welk-O® 1 Kromasil column and 0.1% (v/v) trifluoroacetic acid in isopropanol as organic modifier with the selected conditions (see ***Optimization of the separation conditions*** subsection).

Figure 9S.- Representative SFC-UV chromatograms obtained from a mixture of five urolithin glucuronide standards (100 mg/L; 1.- isourolithin A 9- glucuronide; 2.- isourolithin A 3-glucuronide; 3.- urolithin A 8-glucuronide; 4.- urolithin A 3-glucuronide; 5.- urolithin B 3-glucuronide) using the Reflect™ I-Cellulose C column and 0.1% (v/v) trifluoroacetic acid in different organic solvents: **A**) methanol; **B**) ethanol; **C**) isopropanol (see ***Optimization of the separation conditions*** subsection).

Figure 10S.- Representative SFC-UV chromatograms obtained from a mixture of five urolithin glucuronide standards (100 mg/L; 1.- isourolithin A 9- glucuronide; 2.- isourolithin A 3-glucuronide; 3.- urolithin A 8-glucuronide; 4.- urolithin A 3-glucuronide; 5.- urolithin B 3-glucuronide) using the Reflect™ I-Cellulose C column and different acid additives 0.1% (v/v; **A**) no additive; **B**) formic acid; **C**) acetic acid; **D**) trifluoroacetic acid in methanol (see ***Optimization of the separation conditions*** subsection).

Figure 11S.- Representative SFC-UV chromatograms obtained from a mixture of five urolithin glucuronide standards (100 mg/L; 1.- isourolithin A 9- glucuronide; 2.- isourolithin A 3-glucuronide; 3.- urolithin A 8-glucuronide; 4.- urolithin A 3-glucuronide; 5.- urolithin B 3-glucuronide) using the Reflect™ I-Cellulose C column and different basic additives 0.1% (v/v;

A) no additive; **B**) diethylamine; **C**) trimethylamine; **D**) ethanolamine) in methanol (see ***Optimization of the separation conditions*** subsection)**.**

Figure 1S

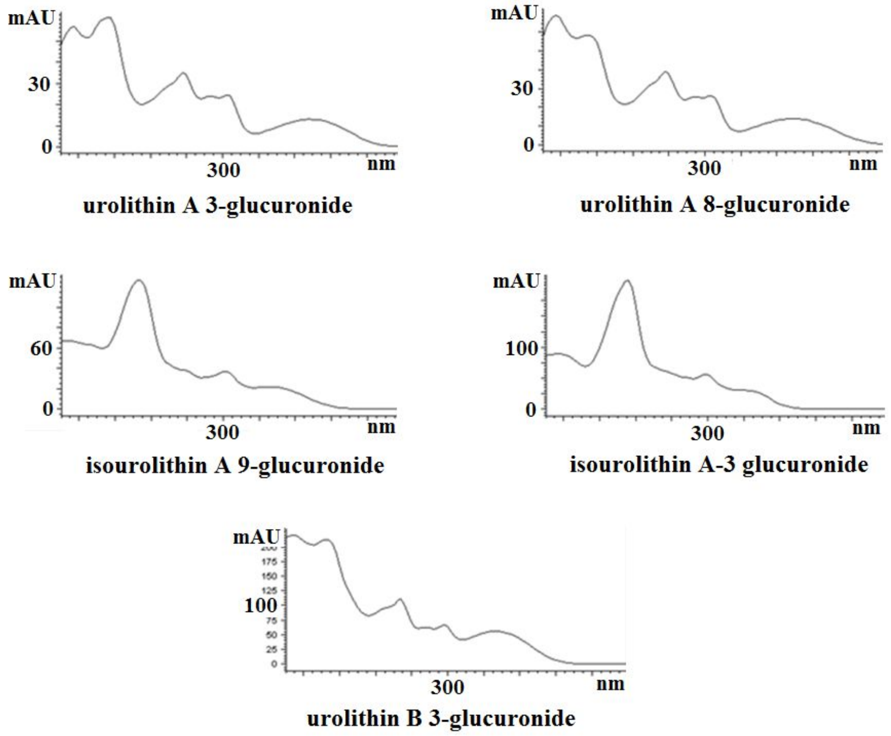
mAU
30
0
300
nm
urolithin A 3-glucuronide
mAU
30
0
300
nm
urolithin A 8-glucuronide
mAU
60
0
300
nm
isourolithin A 9-glucuronide
mAU
100
0
300
nm
isourolithin A-3 glucuronide
mAU
100
0
300
nm
urolithin B 3-glucuronide

Figure 2S

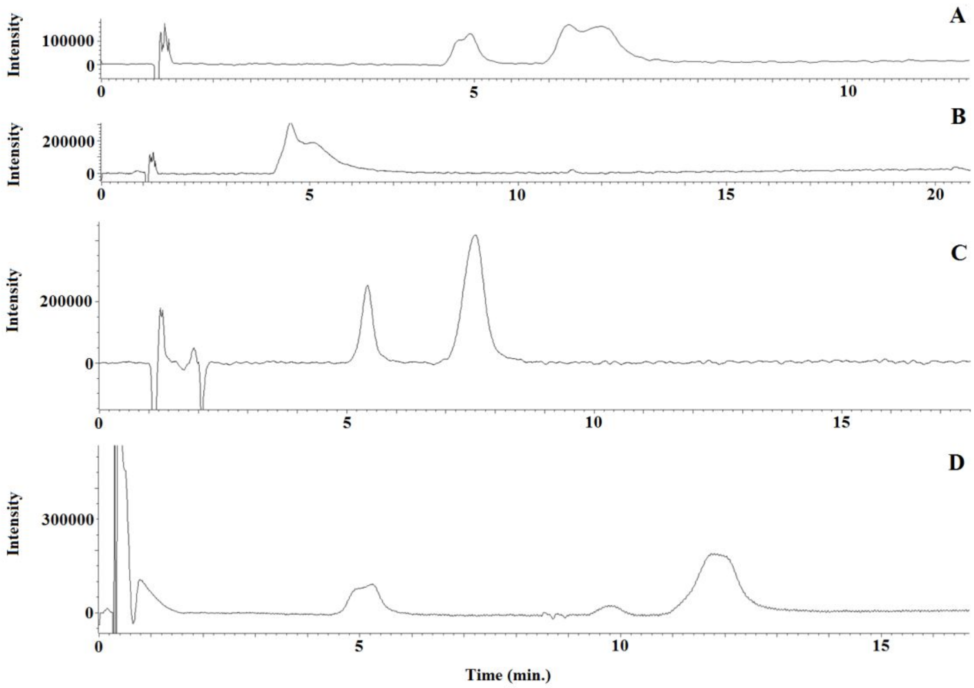

Figure 3S

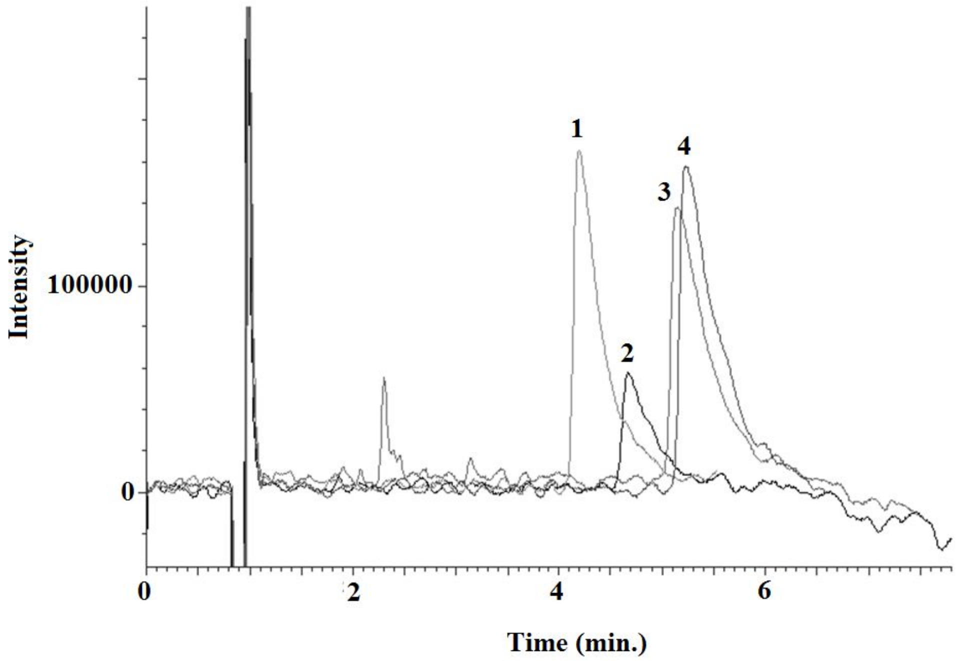

Figure 4S

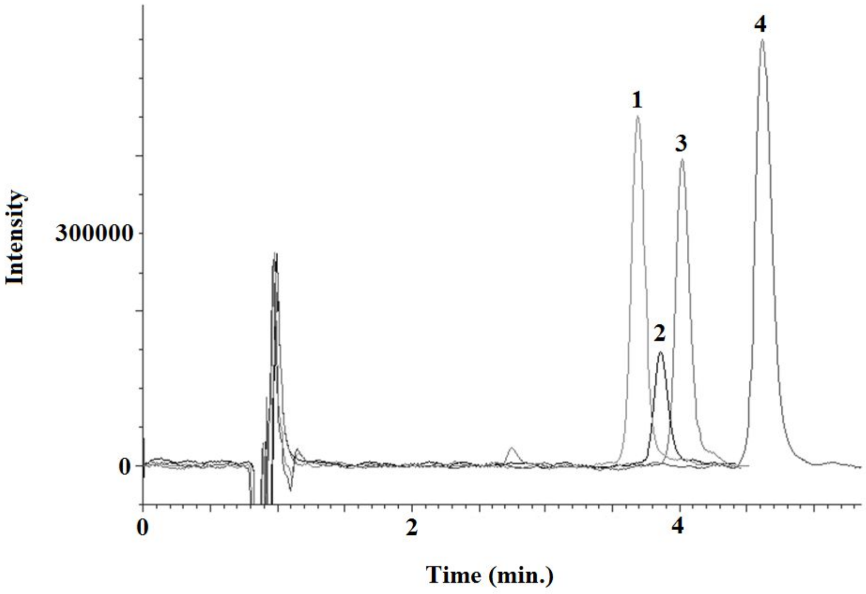

Figure 5S

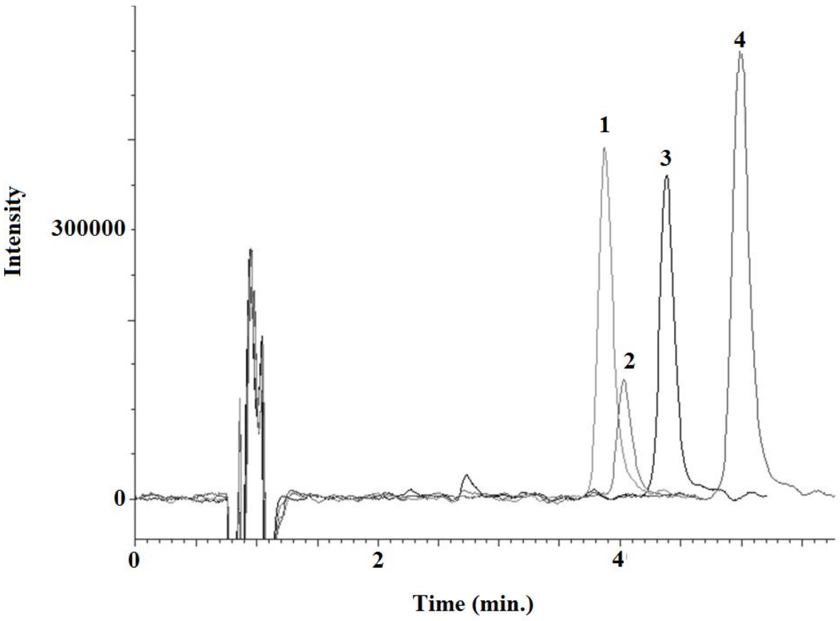

Figure 6S

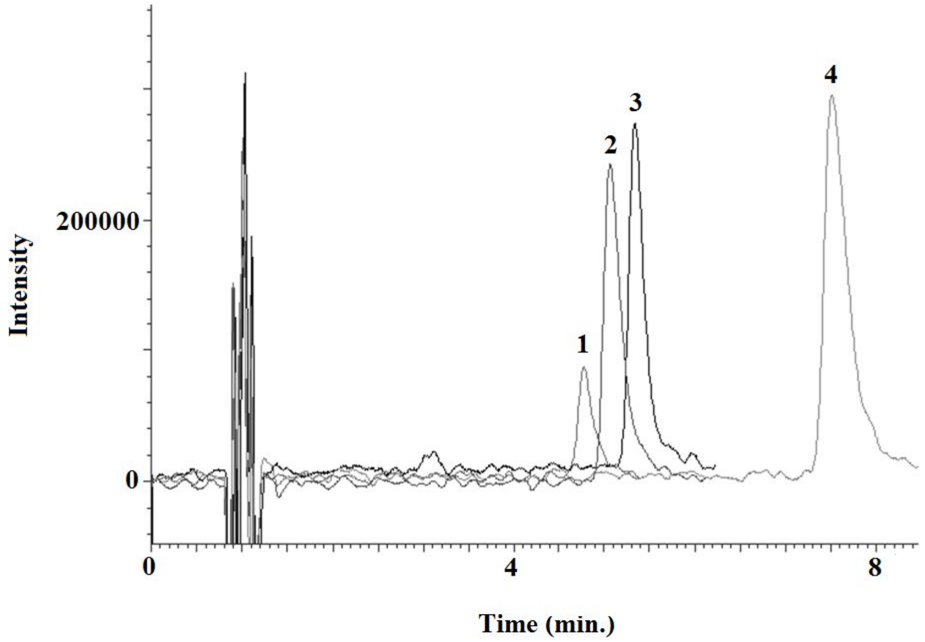

Figure 7S

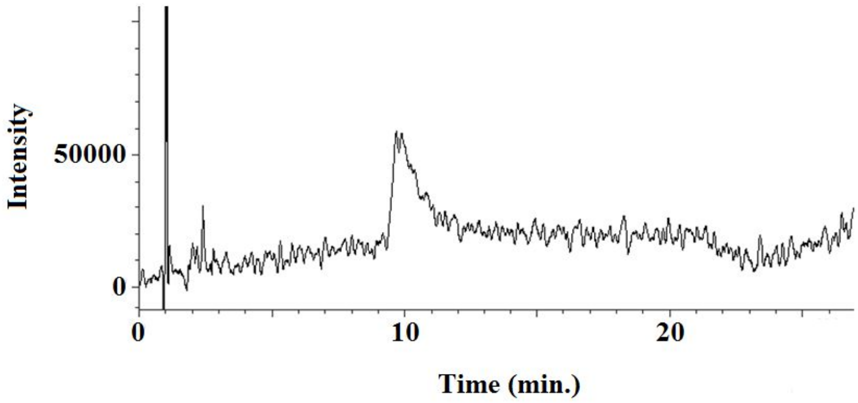

Figure 8S

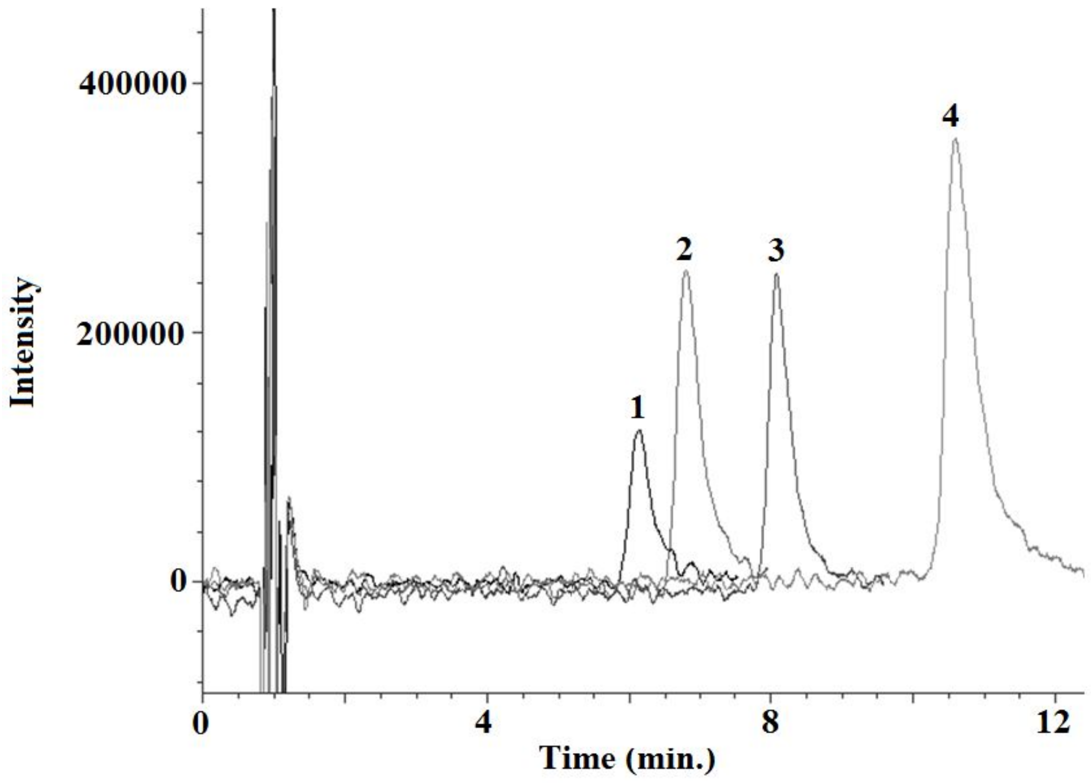

Figure 9S

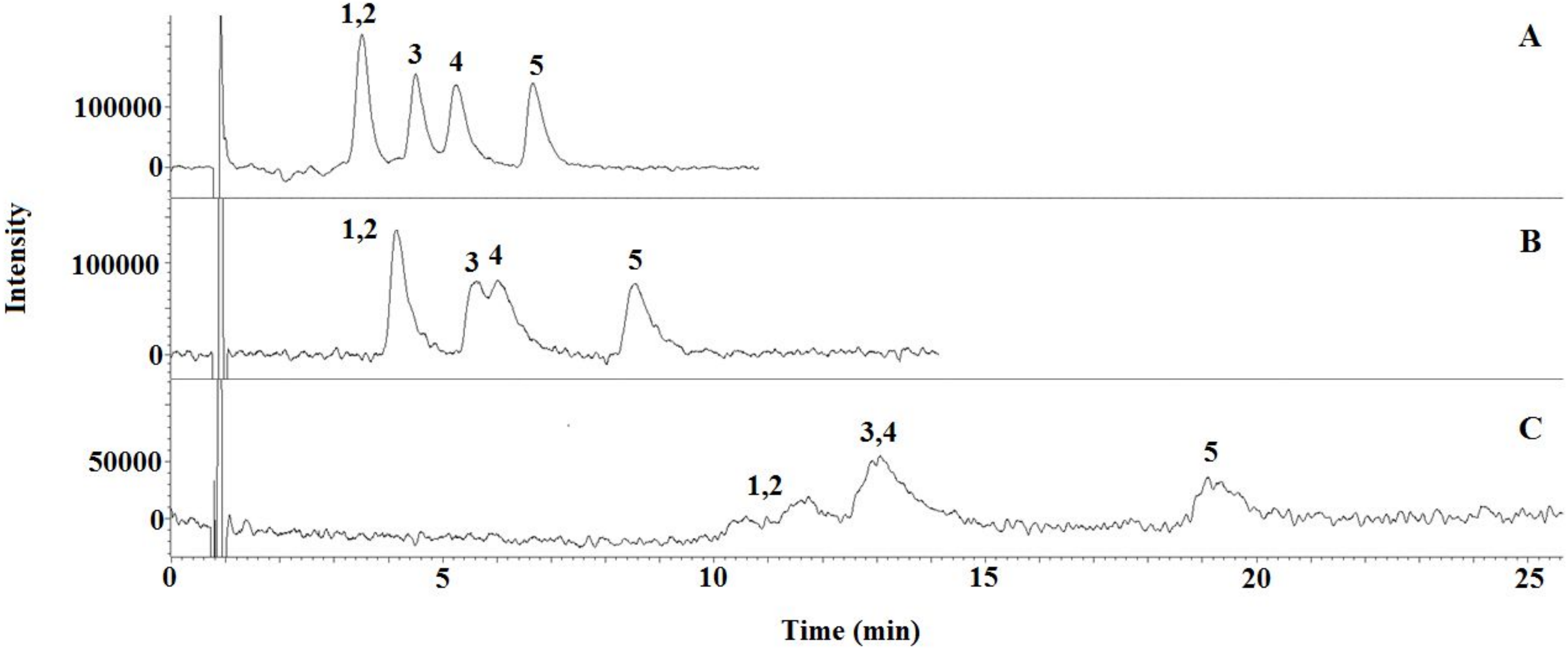

Figure 10S

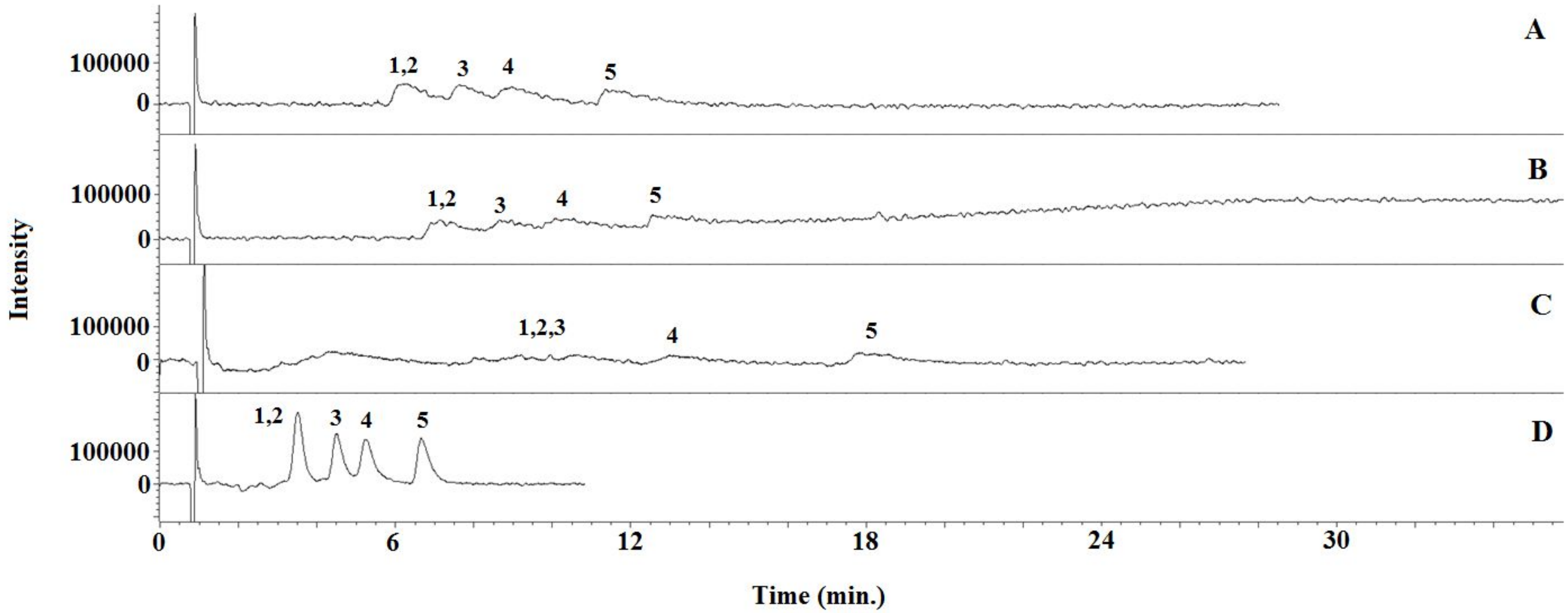

Figure 11S

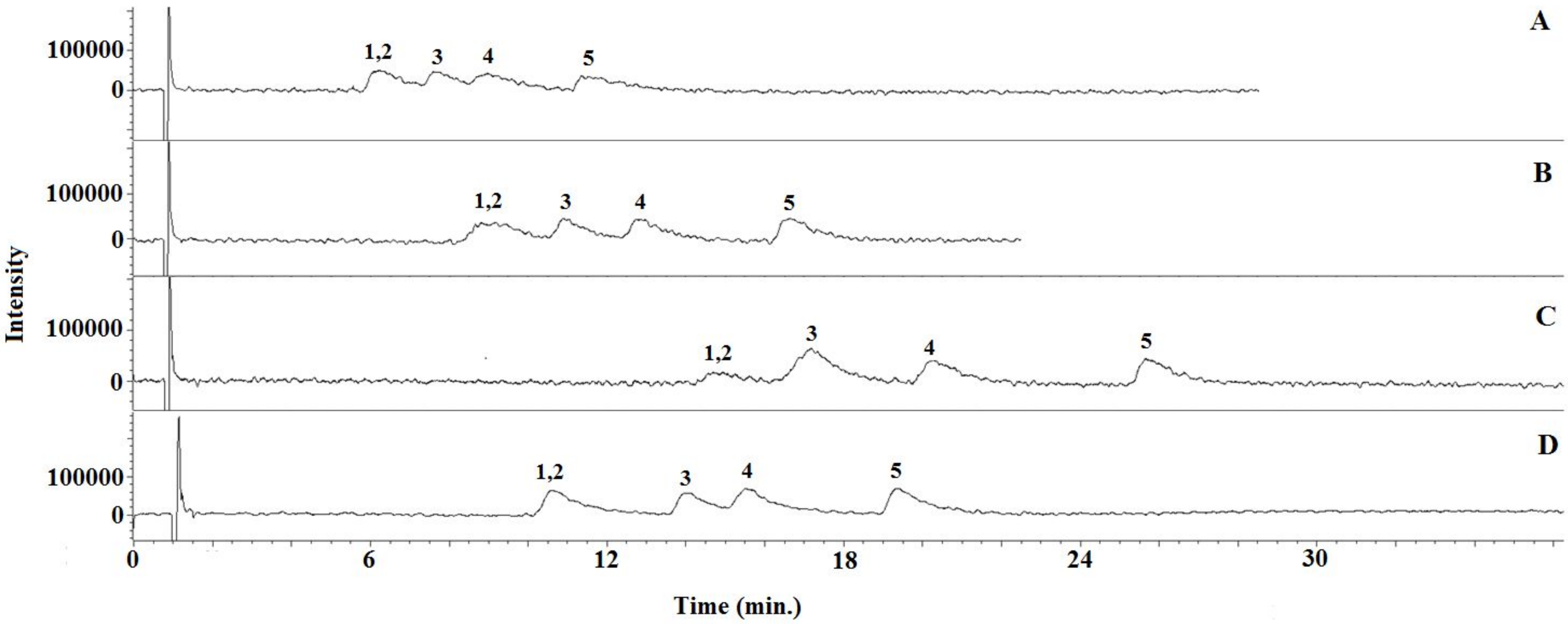

Supplement: Supplementary file 1 — jf2c07145_si_001.pdf [file jf2c07145_si_001.pdf]
